# Dietary Patterns Are Related to Clinical Characteristics in Memory Clinic Patients with Subjective Cognitive Decline: The SCIENCe Project

**DOI:** 10.3390/nu11051057

**Published:** 2019-05-11

**Authors:** Linda M. P. Wesselman, Astrid S. Doorduijn, Francisca A. de Leeuw, Sander C. J. Verfaillie, Mardou van Leeuwenstijn-Koopman, Rosalinde E. R. Slot, Maartje I. Kester, Niels D. Prins, Ondine van de Rest, Marian A. E. de van der Schueren, Philip Scheltens, Sietske A. M. Sikkes, Wiesje M. van der Flier

**Affiliations:** 1 Alzheimer Center Amsterdam, Department of Neurology, Amsterdam Neuroscience, Vrije Universiteit Amsterdam, Amsterdam UMC, 1081HZ Amsterdam, The Netherlands; a.doorduijn@amsterdamumc.nl (A.S.D.); f.deleeuw@vumc.nl (F.A.d.L.); s.verfaillie@vumc.nl (S.C.J.V.); m.vanleeuwenstijn@vumc.nl (M.v.L.-K.); re.slot@vumc.nl (R.E.R.S.); m.kester@vumc.nl (M.I.K.); nd.prins@vumc.nl (N.D.P.); p.scheltens@vumc.nl (P.S.); s.sikkes@vumc.nl (S.A.M.S.); wm.vdFlier@vumc.nl (W.M.v.d.F.); 2 Department of Nutrition and Dietetics, Vrije Universiteit Amsterdam, Amsterdam UMC, 1081HV Amsterdam, The Netherlands; m.devanderschueren@amsterdamumc.nl; 3 Division of Human Nutrition and Health, Wageningen University & Research, 6708WE Wageningen, The Netherlands; ondine.vanderest@wur.nl; 4 Department of Nutrition and Health, HAN University of Applied Sciences, 6525EJ Nijmegen, The Netherlands; 5 Department of Epidemiology and Biostatistics, Amsterdam Neuroscience, Vrije Universiteit Amsterdam, Amsterdam UMC, 1081HV Amsterdam, The Netherlands

**Keywords:** nutrition, cognition, subjective cognitive decline, prevention, Alzheimer’s disease, memory clinic

## Abstract

As nutrition is one of the modifiable risk factors for cognitive decline, we studied the relationship between dietary quality and clinical characteristics in cognitively normal individuals with subjective cognitive decline (SCD). We included 165 SCD subjects (age: 64 ± 8 years; 45% female) from the SCIENCe project, a prospective memory clinic based cohort study on SCD. The Dutch Healthy Diet Food Frequency Questionnaire (DHD-FFQ) was used to assess adherence to Dutch guidelines on vegetable, fruit, fibers, fish, saturated fat, trans fatty acids, salt and alcohol intake (item score 0–10, higher score indicating better adherence). We measured global cognition (Mini Mental State Examination), cognitive complaints (Cognitive Change Index self-report; CCI) and depressive symptoms (Center for Epidemiologic Studies Depression Scale; CES-D). Using principal component analysis, we identified dietary components and investigated their relation to clinical characteristics using linear regression models adjusted for age, sex and education. We identified three dietary patterns: (i) “low-Fat-low-Salt”, (ii) “high-Veggy”, and (iii) “low-Alcohol-low-Fish”. Individuals with lower adherence on “low-Fat-low-Salt” had more depressive symptoms (β −0.18 (−2.27–−0.16)). Higher adherence to “high-Veggy” was associated with higher MMSE scores (β 0.30 (0.21–0.64)). No associations were found with the low-Alcohol-low-Fish component. We showed that in SCD subjects, dietary quality was related to clinically relevant outcomes. These findings could be useful to identify individuals that might benefit most from nutritional prevention strategies to optimize brain health.

## 1. Introduction

Dementia poses a large burden on the health care systems [1,2]. The number of people living with dementia worldwide is estimated to increase from 46 million in 2015, to 131.5 million by 2050 [1] due to aging of the society [2]. In memory clinics, a substantial part of the patients experiences cognitive decline, but does not show any formal deficits on neuropsychological testing, nor any other neurological or psychiatric diagnosis explaining these complaints. This phenomenon is referred to as subjective cognitive decline (SCD) [3]. Previous studies showed that individuals with SCD are at increased risk of developing dementia [4,5]. These individuals are the ideal target population for preventive strategies aimed at promoting brain health.

There is a strong link between lifestyle and cognition, and nutrition has been identified as one of the modifiable risk factors for cognitive decline and dementia [6]. Some studies have shown a relation between consumption of specific foods or food groups and cognitive decline or dementia, such as green leafy vegetables [7], berries [8], or fruit and vegetables [9]. In addition, a growing body of evidence suggests that the quality of the whole diet rather than single nutrients, is key [10,11]. Longitudinal studies have shown benefits of dietary patterns on the development of cognitive impairment, such as the Mediterranean diet (Med-diet; [12,13]), the Dietary Approaches to Stop Hypertension (DASH-diet; [14,15]) and a combination of both (Mediterranean-DASH Intervention for Neurodegenerative Delay (MIND) diet; [16,17]). These three dietary patterns focus on higher consumption of healthy products, such as fruits, nuts, fish, and legumes (e.g., peas, lentils, beans), and limiting the intake of salt, fat and red meat. In scoping reviews, it was suggested that compared to single nutrient interventions, dietary patterns of various nutrients are assumed to exert synergistic effects and are therefore more effective in preventing cognitive decline [18,19].

Most nutritional studies are performed in population-based samples and little is known about the effect of nutritional interventions in at-risk populations. Multi domain lifestyle interventions including a nutritional component targeting at-risk individuals have shown to be beneficial for the prevention of cognitive decline. For example, a large multi-domain intervention called FINGER (Finnish Geriatric Intervention Study to Prevent Cognitive Impairment and Disability) included dietary advice and showed a beneficial effect on cognition in individuals at-risk for cognitive decline based on cardiovascular risk factors [20]. In addition, the Multidomain Alzheimer Preventive Trial (MAPT) evaluated the effects of a multi-domain lifestyle intervention, with or without omega 3 polyunsaturated fatty acids supplements [21]. Exploratory subgroup analyses suggested that the multi-domain intervention might be effective in individuals at most risk for cognitive decline. These studies suggest that lifestyle interventions are particularly effective in individuals at risk for cognitive decline. In the memory clinic, we observe individuals with SCD being eager to know what they can do to maintain their brain health, with a major interest in nutrition [22]. However, little is known about the dietary quality of individuals with SCD and targeted nutritional prevention programs are not available [23].

In a sample of individuals with SCD, we aimed to relate dietary components to clinical characteristics including global cognition, cognitive complaints and depressive symptoms and to compare the demographics of individuals adhering to different dietary patterns.

## 2. Materials and Methods

### 2.1. Participants

In this cross-sectional study, we included 165 participants with SCD from the ongoing SCIENCe project [24]. The SCIENCe project is a prospective memory clinic based cohort study on SCD, with annual follow-up for all participants. For the present study, participants were included when data on dietary quality and clinical characteristics were available. All participants visited the memory clinic of the Alzheimer center Amsterdam for a dementia assessment. They underwent standardized diagnostic work-up, including clinical evaluation, medical history, neuropsychological assessment, physical examination, blood tests, and brain magnetic resonance imaging [25]. Patients were labelled as having SCD when clinical investigations were normal, and clinical criteria for mild cognitive impairment (MCI), dementia or any psychiatric or neurological disorder were not fulfilled. All subjects gave their informed consent for inclusion before they participated in the study. The study was conducted in accordance with the Declaration of Helsinki, and the protocol was approved by the Ethics Committee of the Amsterdam UMC (2014.019).

### 2.2. Dietary Assessment

We used the Dutch Healthy Diet Food Frequency Questionnaire (DHD-FFQ; [26]) to assess averaged daily dietary quality. This questionnaire comprises 34 items that cover eight nutritional items: vegetables, fruit, fibers, fish, saturated fat, trans fatty acids, salt and alcohol. Item scores range from 0 to 10, with the total score ranging from 0 to 80. A higher score indicates a better diet quality, i.e., better adherence to the Dutch guidelines for a Healthy Diet [27,28,29] (Supplement Table S1). For the components, fat, salt and alcohol, a higher score indicates a lower intake. For reference purposes, mean scores from the original validation sample in the general Dutch population (*N* = 1235) are included in Table 1 [30].

We used principal component analysis (PCA) based on the eight nutritional items to identify dietary components (see statistical analysis for details).

### 2.3. Outcome Measures (Clinical Characteristics)

Global cognition was measured with the Mini Mental State Examination (MMSE; [31]), with a maximum score of 30 and a higher score indicating better global cognition. Cognitive complaints were assessed with the self-report Cognitive Change index (CCI [32]); a questionnaire assessing cognitive complaints relative to 5 years ago. The list entails 20 items on a 4 point scale, leading to a maximum score of 80 with a higher score indicating more cognitive complaints. The cutoff value for significant cognitive complaints is set at 16/80. Depressive symptoms were assessed using the Center for Epidemiological Studies-Depression scale (CES-D; [33]). This questionnaire entails 20 items, and each item is scored on a 4 point scale, leading to a maximum score of 60 with a higher score indicating more depressive symptoms. The cutoff value for significant depressive symptoms is set at 16/60.

### 2.4. Statistical Analysis

Analyses were conducted using SPSS version 22 (IBM Corp, New York, NY, USA) [34]. Statistics were presented as mean (sd) or *n* (%) where appropriate. We used PCA (Varimax rotation) based on the eight nutritional items to identify dietary components. Dietary components (factors from the PCA) were extracted based on Eigenvalues greater than 1.

We used linear regression analysis to investigate relationships between nutrition captured by the DHD-FFQ total score and extracted dietary components, and clinical characteristics (CES-D, MMSE, CCI; dependent variables in separate models). Analyses were run unadjusted (model 1) and adjusted for age, sex and education (model 2).

The three empirical-derived dietary components (or PCA factors) are based on the variation between the eight nutritional items of the DHD-FFQ. Each individual has a score on each of the PCA components. Using K-means clustering, we subsequently clustered individuals based on the three PCA components in order to create dietary subgroups. Finally, we compared DHD-FFQ scores, demographic and clinical characteristics between clusters using Chi Squared tests or analysis of variance where appropriate. *p*-values of ≤ 0.05 were considered significant.

## 3. Results

Table 1 presents the demographics, clinical characteristics and nutritional item scores of our sample. Patients (64 ± 8 years old, 45% (*n* = 74) female, 11 ± 5 years of education) scored 29 ± 1 on the MMSE, 44 ± 15 on the CCI and 9 ± 7 on the CES-D. The average DHD-FFQ score was 54 ± 12, with the worst adherence to the guideline for saturated fat (5 ± 4) and best adherence to the alcohol guideline (9 ± 3). Nutritional intake was comparable with the general Dutch population [30]. See Table 1 for nutritional item scores.

Using PCA, we identified three dietary components which together explained 67% of the variance of dietary quality. The factor loadings (Table 2) show that adherence to both fat and salt guidelines positively contributed and adherence to the fiber guideline negatively contributed to dietary component 1 (“low-Fat-low-Salt”). Adherence to vegetable, fruit, fish and fiber guidelines contributed to dietary component 2 (“high-Veggy”). Finally, adherence to the alcohol guideline (low alcohol intake) but not to the fish guideline (low fish intake) loaded on dietary component 3 (“low-Alcohol-low-Fish”).

Using linear regression analysis (Table 3), individuals with lower score on the “low-Fat-low-Salt” component (worse adherence to fat and salt guideline, thus higher fat and salt intake) showed more depressive symptoms on the CES-D (β −0.18 (−2.27–−0.16), *p* < 0.05). In addition, higher scores on the “high-Veggy” component were related to a higher global cognition score on the MMSE (β 0.30 (0.21–0.64), *p* < 0.01). There were no associations with DHD-FFQ total score or the CCI, nor with the low-Alcohol-low-Fish dietary component.

**Figure 1:**
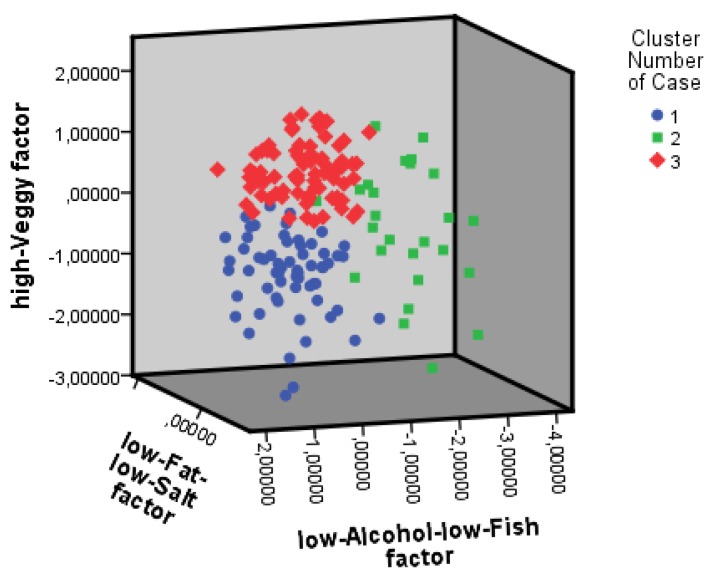
Individuals clustered based on scores on nutritional components. This figure illustrates the three clusters and their scores on the nutritional components (PCA factors). Nutritional components are represented by the axes of the figures. Cluster 1: blue; Cluster 2: green; Cluster 3: red.

Subsequently, we clustered individuals using K-means cluster analysis based on the three dietary components (Figure 1; see Supplement Table S2 for cluster centers). Distinction between the clusters is mainly based on differences on “high-Veggy” component scores, complemented with scores on the low-Alcohol-low-Fish component. Adherence to saturated fat, trans fatty acids and salt guidelines did not differ between clusters. Clusters differed in total DHD-FFQ score, with Cluster 3 having a higher score than Cluster 1, and Cluster 2 having a score in between Cluster 1 and 3. Cluster 3 adhered best to the guidelines for vegetable intake compared to the other clusters. With regard to fruit and fiber intake, Cluster 3 adhered better to the guidelines than Cluster 1 and 2. The adherence to fish guideline of Cluster 1 was low compared to Cluster 2 and 3. Cluster 2 adhered worst to the alcohol guidelines, compared to Clusters 1 and 3.

ANOVA’s showed that clusters differed in sex and education and adjusted for age, sex and education also differed in cognitive complaints (CCI) (Table 4). The proportion of females were higher in Cluster 2 than Cluster 1 and 3. Individuals in Cluster 3 had higher level of education compared to individuals in Cluster 1. In addition, individuals from Cluster 1 had a higher CCI, i.e., reported more cognitive complaints compared to individuals in Cluster 2. Clusters did not differ in MMSE or CES-D.

## 4. Discussion

The main findings of the current study in individuals with SCD at a memory clinic were that better adherence to a “high-Veggy” dietary component was related to better global cognition, while lower adherence to a “low-Fat-low-Salt” dietary component (i.e., higher consumption of fat and salt) was related to more depressive symptoms. In addition, individuals with the unhealthiest diet with lowest adherence to fruit and vegetable guidelines reported most cognitive complaints. These findings suggest that even in cognitively normal elderly at risk of decline, nutritional intake is related to clinically relevant characteristics.

In this study we used a data driven clustering method to derive patterns from the dietary intake of the participants. Our results extend on earlier findings that show beneficial effects of predefined patterns such as the Med-diet, DASH-diet and MIND-diet on cognitive function [10,16]. Regarding subjective cognitive functioning, a beneficial effect of both adherence to the Med-diet [35] as well as long-term intake of vegetable, fruit and orange juice [36] was found in a large male population-based study. The Australian Imaging, Biomarker & Lifestyle Study of Ageing (AIBL) study [37] found that adherence to the Med-diet was associated with clinical progression to MCI or dementia in initially non-demented, without taking SCD into account.

Although we did not have completely the same nutritional components as the Med-, MIND- and DASH diets, we do overlap in recommendations of high intake of fruits, vegetables, fish and fibers and limiting intake of salt and fat. We add relevant knowledge by showing that dietary quality is related to clinical characteristics, amongst other global cognition. 

A higher score on the “high-Veggy” component was related to a higher global cognition score. In addition, in this population without clinical depression, individuals not adhering to the guidelines for fat and salt, showed more depressive symptoms, which is in accordance with literature from population-based studies [7,38].

When individuals were clustered based on dietary quality, they differed in cognitive complaints, education and sex. These findings are relevant for identifying target groups that might benefit most from prevention and can be used to select specific dietary components for interventions. While not all SCD patients will progress to dementia, they do present themselves at memory clinics with complaints and questions. Part of this patient group has subclinical psychiatric symptoms [24], while others might be worried-well and again other will indeed progress to dementia. This study emphasizes that nutrition is an important target for brain health and well-being in this clinically relevant group.

There is no literature available on the relationship between nutrition and cognition in subjects with SCD, as current literature on the relation between nutrition and cognitive decline invariably resulted from population-based studies [39]. Individuals with SCD presenting at the memory clinic are eager to learn about the relevance of nutrition and lifestyle to promote their own brain health [22]. From an ongoing multi-domain lifestyle intervention in older adults at risk of dementia, we know that diet improves after nutritional advice, resulting in beneficial effects on cognitive function [40,41]. Therefore, subjects with SCD are an interesting and promising target group for dietary advice to prevent cognitive decline.

The current study was performed in a clinical population with SCD and therefore contributes to bridging the gap between cognitive healthy older adults and patients with dementia. We included individuals that are part of a well-phenotyped prospective cohort study of subjective cognitive decline [24]. We follow these individuals annually, which gives us a unique opportunity to identify possible nutrition-related cognitive decline over time. The results could be useful for the implementation of a nutrition-based intervention program.

This study has some limitations. The cross-sectional design allows us to generate hypotheses for future research, rather than draw conclusions about the causality of the associations that were found. In this study, linear regression analysis was used. Another interesting aspect would be to investigate whether a dose-response or non-linear relationship is present for vegetable and low salt and fat intake. In a recent meta-analysis, a dose-response relationship was demonstrated for vegetable intake and risk for cognitive impairment and dementia [42]. We could not address this relationship, as we did not measure nutritional intake in absolute quantities, and this would therefore be interesting to explore in future studies.

The DHD-FFQ assesses dietary quality and not quantity, includes a limited number of food items and does not take nutritional supplement use into account. Also, specific dietary patterns or restrictions could influence the DHD-FFQ scores. However, the DHD-FFQ is an easy-to-use validated short questionnaire [30], useful for the assessment of diet quality in a large group of individuals in a clinical research setting. The estimated energy intake coverage of the DHD-FFQ is 64% [30]. To assess full dietary intake, a more extensive but also more time-consuming method such as a food frequency questionnaire or a nutritional intake diary could be useful. The DHD-FFQ measures adherence to the Dutch guidelines for a Healthy Diet, but these are in line with the World Health Organization for a healthy diet [43].

This explorative cross-sectional study showed that there is a relation between dietary quality and clinical characteristics in individuals with SCD in a memory clinic setting. Better adherence to fruit and vegetable guidelines was related to better cognitive performance, and lower adherence to fat and salt guidelines was related to more depressive symptoms. SCD individuals present themselves at memory clinics with a need for help but a diversity of complaints and future progression. Clinical characteristics might be useful to identify target groups that might benefit most personalized nutritional interventions in order to gain maximum beneficial effects for brain health.

## Figures and Tables

**Table 1:** Nutritional item scores of the total group.

Nutritional Item (Mean ± SD)	Total SCD Group (*N* = 165)	General Dutch Population [30]
DHD-FFQ total score	54.3 (12.2)	57.6 (9.6)
Vegetables	6.6 (2.9)	6.7 (2.6)
Fruit	7.8 (2.8)	8.0 (2.7)
Fibers	7.5 (1.9)	7.8 (1.9)
Fish	6.2 (3.4)	5.5 (3.2)
Saturated fat *	4.5 (4.1)	5.5 (4.0)
Trans fatty acids *	7.3 (4.6)	9.2 (2.7)
Salt *	5.9 (3.0)	6.3 (2.8)
Alcohol *	8.7 (2.7)	8.6 (2.7)

This table presents the nutritional item scores of the total group. Although the score on trans fatty acids could be 0 or 10 (yes/no adherence) the score was treated as a continuous variable. In order to put these numbers into context, the results of a study in the Dutch population were added in the right column. * A higher score indicates better adherence to the nutritional guideline, and therefore a lower quantitative intake in this category. DHD-FFQ: Dutch Healthy Diet Food Frequency Questionnaire.

**Table 2:** PCA components: Factor loadings.

Nutritional Item	Component 1	Component 2	Component 3
Saturated fat	0.837	0.004	−0.031
Salt	0.821	−0.031	0.025
Trans fatty acids	0.807	0.094	0.010
Vegetables	−0.042	0.792	−0.137
Fruit	0.205	0.718	0.129
Fibers	−0.566	0.649	0.098
Alcohol	−0.017	0.172	0.868
Fish	−0.003	0.457	−0.581

This table presents the factor loadings of the nutritional items on the dietary components that were identified using principal component analysis. PCA: principal component analysis.

**Table 3:** Linear regression models of PCA components and clinical characteristics.

Nutritional Score	Model	MMSE	CCI	CES-D
DHD-FFQ Total Score	1	β 0.10 (−0.01–0.03)	β −0.01 (−0.21–0.18)	β −0.11 (−0.15–0.02)
	2	β 0.12 (−0.01.–0.03)	β 0.03 (−0.16–0.24)	β −0.13 (−0.16–0.02)
“Low-Fat-low-Salt” Component	1	β −0.10 (−0.36–0.08)	β −0.01 (−2.5–2.2)	β −0.17 * (−2.1–−0.10)
	2	β −0.10 (−0.36–0.09)	β 0.04 (−1.8–3.0)	β −0.18 * (−2.27–−0.16)
“high-Veggy” Component	1	β 0.27 ** (0.17–0.59)	β −0.04 (−2.9–1.8)	β 0.03 (−0.82–1.25)
	2	β 0.30 ** (0.21–0.64)	β −0.02 (−2.7–2.0)	β 0.02 (−0.91–1.23)
“Low-Alcohol-low-Fish” Component	1	β −0.09 (−0.35–0.09)	β 0.15 (−0.12–4.5)	β 0.01 (−0.99–1.08)
	2	β −0.10 (−0.37–0.08)	β 0.12 (−0.60–4.1)	β 0.01 (−0.98–1.13)

This table presents the results of linear regression models between the DHD-FFQ total score and PCA components, and the clinical characteristics. Reported are the standardized beta coefficients and (95% confidence interval). Model 1: uncorrected. Model 2: corrected for age, sex and education. * *p* < 0.05, ** *p* < 0.01. PCA: principal component analysis, MMSE: Mini-Mental State Examination, CCI: Cognitive Change Index, CES-D: Center for Epidemiologic Studies Depression scale, DHD-FFQ: Dutch Healthy Diet Food Frequency Questionnaire.

**Table 4:** Group comparisons between the 3 clusters.

Characteristic Mean (SD)	Cluster 1 *n* = 61	Cluster 2 *n* = 26	Cluster 3 *n* = 78
Female, *n* (%)	22 (36%) ^&^	18 (69%) ^#^	34 (44%) ^&#^
Age, year	62.3 (8.0)	63.9 (8.4)	65.1 (7.9)
Education, year	9.1 (4.9) ^&^	11.2 (5.6)	11.3 (4.8) ^&^
BMI (kg/m^2^)	26 (4)	27 (4)	25 (3)
Smoker ^a^: *n* (%)			
no	23 (37%)	13 (50%)	40 (51%)
previous	27 (44%)	11 (42%)	31 (40%)
current	9 (15%)	1 (4%)	3 (4%)
DHD-FFQ total score	48.0 (10.4) ^#^	50.5 (11.9)	61.2 (9.6) ^#^
Vegetables	4.0 (2.1) ^#^	7.0 (2.5) ^#^	8.5 (1.8) ^#^
Fruit	6.1 (3.1) ^#^	7.0 (3.0) ^&^	9.4 (1.2) ^#&^
Fibers	6.4 (1.8) ^#^	7.0 (1.9) ^&^	8.4 (1.5) ^#&^
Fish	4.5 (3.2) ^#^	8.2 (2.8) ^#&^	7.0 (3.2) ^&^
Saturated fat *	4.1 (4.2)	4.9 (4.2)	4.8 (4.0)
Trans fatty acids * adherence yes, *n* (%)	40 (66%)	18 (69%)	58 (74%)
Salt *	5.6 (3.3)	6.2 (2.9)	5.9 (2.9)
Alcohol *	9.7 (0.9) ^#^	3.4 (2.9) ^#&^	9.7 (1.0) ^&^
MMSE	28.3 (1.8)	28.8 (1.4)	28.8 (1.0)
CCI	47.1 (15.9) ^&^	38.5 (12.7) ^&^	42.4 (14.9)
CES-D	9.6 (6.9)	9.7 (6.9)	9.0 (6.5)

This table presents the demographics, nutritional intake scores and clinical characteristics of the three clusters. Individuals were clustered based on their scores on the nutritional component (PCA factors). Groups comparison were conducted using Chi Square tests or analysis of variance, ^&,#^ *p* < 0.01. *: higher score indicates better adherence to the nutritional guideline, and therefore a lower quantitative intake in this category. ^a^: Information on smoking was available for 158/165 participants. BMI: body mass index, DHD-FFQ: Dutch Healthy Diet Food Frequency Questionnaire, MMSE: Mini-Mental State Examination, CCI: Cognitive Change Index, CES-D: Center for Epidemiologic Studies Depression scale.

## Supplementary Materials

The following are available online at https://www.mdpi.com/2072-6643/11/5/1057/s1, Table S1: Dutch guidelines for a Healthy Diet, Table S2: Cluster centers based on PCA components.
