# Supplementary material for: Dietary Patterns Are Related to Clinical Characteristics in Memory Clinic Patients with Subjective Cognitive Decline: The SCIENCe Project
Source: Nutrients. 2019 May 11;11(5):1057. doi: 10.3390/nu11051057 (PMC6566666; doi:10.3390/nu11051057)
Supplement: Supplementary file 1 [file nutrients-11-01057-s001.pdf]

Supplement Table S1: Dutch guidelines for a Healthy Diet.

Components		Minimum score (=0)	Maximum score (=10)
1.	Physical activity (week)	0 activities	≥ 5 activities
2.	Vegetable (day)	0 g	≥200 g
3.	Fruit + fruit juices (day)*	0 g	≥ 200 g
4.	Fiber (day)	0 g/4.2MJ	≥14 g/4.2MJ
5.	Fish (day)†	0 mg EPA+DHA	≥ 450 mg EPA+DHA
6.	SFA (day)	≥ 16.6 en%	< 10 en%
7.	TFA (day)	≥ 1.6 en%	< 1 en%
8.	ADF (day)‡	> 7 occasions	≤ 7 occasions
9.	Sodium (day)	≥ 2.45 g	< 1.68 g
10.	Alcohol (day)	♂: ≥ 60 g<br>♀: ≥ 40 g	♂: ≤ 20 g<br>♀: ≤ 10 g

SFA: saturated fatty acids, TFA: trans fatty acids, ADF acidic drinks and foods
*Maximum of 100 gram of juice could be included
†EPA and DHA intake form foods and fish oil capsules
‡The number of consumption ocassions was defined as the number of hours where at least one food or drink with a pH<5.5 and total acidity>0.5 was consumed

NOTE: This Table presents the original guidelines of the DHD-FFQ. In the present study, physical activity and occasions of acidic drinks and foods were not included.

9. References 47/50

[1] Prince M, Wimo A, Guerchet M, Ali GC, Wu YT, Prina M, et al. World Alzheimer Report 2015. The Global Impact of Dementia: An analysis of prevalence, incid ence, cost and trends. World Alzheimer Report. London.: Alzheimer's Disease International (ADI); 2015.
[2] Prince M, Ali GC, Guerchet M, Prina AM, Albanese E, Wu YT. Recent global trends in the prevalence and incidence of dementia, and survival with dementia. Alzheimers Res Ther. 2016;8:23.
[3] Jessen F, Amariglio RE, van Boxtel M, Breteler M, Ceccaldi M, Chetelat G, et al. A conceptual framework for research on subjective cognitive decline in preclinical Alzheimer's disease. Alzheimers Dement. 2014;10:844-52.
[4] van Harten AC, Mielke MM, Swenson-Dravis DM, Hagen CE, Edwards KK, Roberts RO, et al. Subjective cognitive decline and risk of MCI: The Mayo Clinic Study of Aging. Neurology. 2018;91:e300-e12.
[5] Slot RER, Sikkes SAM, Berkhof J, Brodaty H, Buckley R, Cavedo E, et al. Subjective cognitive decline and rates of incident Alzheimer's disease and non-Alzheimer's disease dementia. Alzheimer's & Dementia. 2018;15:465-76.
[6] Livingston G, Sommerlad A, Orgeta V, Costafreda SG, Huntley J, Ames D, et al. Dementia prevention, intervention, and care. Lancet. 2017;pii: S0140-6736:31363-6.
[7] Morris MC, Evans DA, Tangney CC, Bienias JL, Wilson RS. Associations of vegetable and fruit consumption with age-related cognitive change. Neurology. 2006;67:1370-6.
[8] Morris MC, Wang Y, Barnes LL, Bennett DA, Dawson-Hughes B, Booth SL. Nutrients and bioactives in green leafy vegetables and cognitive decline: Prospective study. Neurology. 2018;90:e214-e22.
[9] Kang JH, Ascherio A, Grodstein F. Fruit and vegetable consumption and cognitive decline in aging women. Ann Neurol. 2005;57:713-20.

[10] Fischer K, Melo van Lent D, Wolfsgruber S, Weinhold L, Kleineidam L, Bickel H, et al. Prospective Associations between Single Foods, Alzheimer's Dementia and Memory Decline in the Elderly. Nutrients. 2018;10:852.
[11] Dominguez LJ, Barbagallo M. Nutritional prevention of cognitive decline and dementia. Acta Biomed. 2018;89:276-90.
[12] van de Rest O, Berendsen AA, Haveman-Nies A, de Groot LC. Dietary patterns, cognitive decline, and dementia: a systematic review. Adv Nutr. 2015;6:154-68.
[13] McEvoy CT, Guyer H, Langa KM, Yaffe K. Neuroprotective Diets Are Associated with Better Cognitive Function: The Health and Retirement Study. J Am Geriatr Soc. 2017;65:1857-62.
[14] Lourida I, Soni M, Thompson-Coon J, Purandare N, Lang IA, Ukoumunne OC, et al. Mediterranean Diet, Cognitive Function, and Dementia: A Systematic Review. Epidemiology. 2013;24:479-89.
[15] Singh B, Parsaik AK, Mielke MM, Erwin PJ, Knopman DS, Petersen RC, et al. Association of mediterranean diet with mild cognitive impairment and Alzheimer's disease: a systematic review and meta-analysis. J Alzheimers Dis. 2014;39:271-82.
[16] Wengreen H, Munger RG, Cutler A, Quach A, Bowles A, Corcoran C, et al. Prospective study of Dietary Approaches to Stop Hypertension- and Mediterranean-style dietary patterns and age-related cognitive change: the Cache County Study on Memory, Health and Aging. Am J Clin Nutr. 2013;98:1263-71.
[17] Berendsen AAM, Kang JH, van de Rest O, Feskens EJM, de Groot L, Grodstein F. The Dietary Approaches to Stop Hypertension Diet, Cognitive Function, and Cognitive Decline in American Older Women. J Am Med Dir Assoc. 2017;18:427-32.
[18] Morris MC, Tangney CC, Wang Y, Sacks FM, Barnes LL, Bennett DA, et al. Mind diet slows cognitive decline with aging. Alzheimers Dement. 2015;11:1015-22.
[19] Berendsen AM, Kang JH, Feskens EJM, de Groot C, Grodstein F, van de Rest O. Association of Long-Term Adherence to the MIND Diet with Cognitive Function and Cognitive Decline in American Women. J Nutr Health Aging. 2018;22:222-9.
[20] Abbatecola AM, Russo M, Barbieri M. Dietary patterns and cognition in older persons. Curr Opin Clin Nutr Metab Care. 2018;21:10-3.
[21] Vauzour D, Camprubi-Robles M, Miquel-Kergoat S, Andres-Lacueva C, Banati D, Barberger-Gateau P, et al. Nutrition for the ageing brain: Towards evidence for an optimal diet. Ageing Res Rev. 2017;35:222-40.
[22] Ngandu T, Lehtisalo J, Solomon A, Levälahti E, Ahtiluoto S, Antikainen R, et al. A 2 year multidomain intervention of diet, exercise, cognitive training, and vascular risk monitoring versus control to prevent cognitive decline in at-risk elderly people (FINGER): a randomised controlled trial. The Lancet. 2015;385:2255-63.
[23] Andrieu S, Guyonnet S, Coley N, Cantet C, Bonnefoy M, Bordes S, et al. Effect of long-term omega 3 polyunsaturated fatty acid supplementation with or without multidomain intervention on cognitive function in elderly adults with memory complaints (MAPT): a randomised, placebo-controlled trial. The Lancet Neurology. 2017;16:377-89.
[24] Wesselman LMP, Schild A-K, Coll-Padros N, van der Borg WE, Meurs JHP, Hooghiemstra AM, et al. Wishes and preferences for an online lifestyle program for brain health—A mixed methods study. Alzheimer's & Dementia: Translational Research & Clinical Interventions. 2018;4:141-9.
[25] Smart CM, Karr JE, Areshenkoff CN, Rabin LA, Hudon C, Gates N, et al. Non-Pharmacologic Interventions for Older Adults with Subjective Cognitive Decline: Systematic Review, Meta-Analysis, and Preliminary Recommendations. Neuropsychol Rev. 2017;27:245-57.
[26] Slot RER, Verfaillie SCJ, Overbeek JM, Timmers T, Wesselman LMP, Teunissen CE, et al. Subjective Cognitive Impairment Cohort (SCIENCe): study design and first results. Alzheimers Res Ther. 2018;10:76.
[27] van der Flier WM, Scheltens P. Amsterdam Dementia Cohort: Performing Research to Optimize Care. J Alzheimers Dis. 2018;62:1091-111.

[28] van Lee L, Geelen A, Hooft van Huysduynen EJC, de Vries JHM, van't Veer P, Feskens EJM. The Dutch Healthy Diet index (DHD-index): an instrument to measure adherence to the Dutch guidelines for a healthy diet. Nutrition Journal. 2012;11.
[29] Guidelines for a healthy diet 2006. Publication no 2006/21. The Hague: Health Council of the Netherlands; 2006.
[30] Guidelines for a healthy diet 2006. Background document. publication no A06/08. The Hague: Health Council of the Netherlands; 2006.
[31] The Netherlands Nutrition Centre http://www.voedingscentrum.nl.
[32] van Lee L, Feskens EJ, Meijboom S, Hooft van Huysduynen EJ, van't Veer P, de Vries JH, et al. Evaluation of a screener to assess diet quality in the Netherlands. Br J Nutr. 2016;115:517-26.
[33] Folstein M. "Mini-mental state". A practical method for grading the cognitive state of patients for the clinician. J Psychiatr Res. 1975;12.
[34] Saykin AJ, Wishart HA, Rabin LA, Santulli RB, Flashman LA, West JD, et al. Older adults with cognitive complaints show brain atrophy similar to that of amnestic MCI. Neurology 2006;67.
[35] The CES-D Scale: A Self-Report Depression Scale for Research in the General Population. Appl Psychol Meas. 1977;1:385–401.
[36] IBM. IBM SPSS Statistics for Windows. version 22.0 ed. New York: IBM Corp; 2011.
[37] Solfrizzi V, Custodero C, Lozupone M, Imbimbo BP, Valiani V, Agosti P, et al. Relationships of Dietary Patterns, Foods, and Micro- and Macronutrients with Alzheimer's Disease and Late-Life Cognitive Disorders: A Systematic Review. J Alzheimers Dis. 2017;59:815-49.
[38] Greenwood CE, Parrott MD. Nutrition as a component of dementia risk reduction strategies. Healthc Manage Forum. 2017;30:40-5.
[39] Scarmeas N, Anastasiou CA, Yannakoulia M. Nutrition and prevention of cognitive impairmen. the Lancet Neurology. 2018;17:1006-15.
[40] Morris MC. Nutrition and risk of dementia: overview and methodological issues. Ann N Y Acad Sci. 2016;1367:31-7.
[41] Bhushan A, Fondell E, Ascherio A, Yuan C, Grodstein F, Willett W. Adherence to Mediterranean diet and subjective cognitive function in men. Eur J Epidemiol. 2018;33:223-34.
[42] Gardener S, Gu Y, Rainey-Smith SR, Keogh JB, Clifton PM, Mathieson SL, et al. Adherence to a Mediterranean diet and Alzheimer's disease risk in an Australian population. Transl Psychiatry. 2012;2:e164.
[43] Lehtisalo J, Ngandu T, Valve P, Antikainen R, Laatikainen T, Strandberg T, et al. Nutrient intake and dietary changes during a 2-year multi-domain lifestyle intervention among older adults: secondary analysis of the Finnish Geriatric Intervention Study to Prevent Cognitive Impairment and Disability (FINGER) randomised controlled trial. Br J Nutr. 2017;118:291-302.
[44] Rosenberg A, Ngandu T, Rusanen M, Antikainen R, Backman L, Havulinna S, et al. Multidomain lifestyle intervention benefits a large elderly population at risk for cognitive decline and dementia regardless of baseline characteristics: The FINGER trial. Alzheimers Dement. 2018;14:263-70.
[45] Cabout M, Brouwer IA, Visser M. The MooDFOOD project: Prevention of depression through nutritional strategies. Nutrition Bulletin. 2017;42:94-103.
[46] Looman M, Feskens EJ, de Rijk M, Meijboom S, Biesbroek S, Temme EH, et al. Development and evaluation of the Dutch Healthy Diet index 2015. Public health nutrition. 2017;20:2289-99.
[47] Healthy diet. In: Organization WH, editor. https://wwwwhoint/news-room/fact-sheets/detail/healthy-diet2018.

Supplement Table S2: Cluster centers based on PCA components.

PCA component	Cluster 1	Cluster 2	Cluster 3
1 (Fat-Salt)	-,08058	,05939	,04322
2 (Veggy)	-,91458	-,26624	,80399
3 (low-Alcohol-low-Fish)	,48385	-1,86996	,24492

NOTE: This Table presents the cluster centers of the three PCA components. The PCA components are based on the DHD-FFQ items.
